# Improving outcomes for people who are homeless and have severe mental illness in Ethiopia, Ghana and Kenya: overview of the HOPE programme

**DOI:** 10.1017/S2045796025000186

**Published:** 2025-04-21

**Authors:** Charlotte Hanlon, Caroline Smartt, Victoria N. Mutiso, Peter Yaro, Eleni Misganaw, Ursula Read, Rosie Mayston, Ribka Birhanu, Phyllis Dako-Gyeke, David M. Ndetei, Laura Asher, Julie Repper, Julian Eaton, Kia–Chong Chua, Abebaw Fekadu, Ruth Tsigebrhan, Cecilia Ashaley Fofo, Kimberly Kariuki, Sauharda Rai, Sisay Abayneh, Caroline Reindorf Amissah, Amma Mpomaa Boadu, Priscilla Makau, Agitu Tadesse, Philip Timms, Martin Prince, Graham Thornicroft, Brandon Kohrt, Atalay Alem

**Affiliations:** 1 Division of Psychiatry, Centre for Clinical Brain Sciences, University of Edinburgh, Edinburgh, UK; 2 Centre for Innovative Drug Development and Therapeutic Trials for Africa (CDT-Africa), College of Health Sciences, Addis Ababa University, Addis Ababa, Ethiopia; 3 Centre for Global Mental Health, Health Service and Population Research Department, Institute of Psychiatry, Psychology and Neuroscience, King’s College London, London, UK; 4 Africa Institute of Mental and Brain Health, Nairobi, Kenya; 5 BasicNeeds-Ghana, Tamale, Ghana; 6 Mental Health Service User Association, Addis Ababa, Ethiopia; 7 School of Health and Social Care, University of Essex, Colchester, UK; 8 Department of Global Health & Social Medicine, King’s College London, London, UK; 9 College of Health Sciences, School of Medicine, Department of Psychiatry and WHO Collaborating Centre in Mental Health Research and Capacity-Building, Addis Ababa University, Addis Ababa, Ethiopia; 10 Department of Social and Behavioural Sciences, School of Public Health, University of Ghana, Accra, Ghana; 11 Department of Psychiatry, University of Nairobi, Nairobi, Kenya; 12 Centre for Public Health and Epidemiology, School of Medicine, University of Nottingham, Nottingham, UK; 13 Institute of Mental Health, University of Nottingham, Nottingham, UK; 14 Imroc (Charity Reg No. 1207904), Nottingham, UK; 15 CBM Global, UK; 16 Department of International Public Health, Liverpool School of Tropical Medicine, Liverpool, UK; 17 Department of Biostatistics and Health Informatics, Institute of Psychiatry, Psychology, and Neuroscience, King’s College London, London, UK; 18 Department of Global Health & Infection, Brighton and Sussex Medical School, Brighton, UK; 19 BasicNeeds-Ghana, Accra, Ghana; 20 Centre for Clinical Research, Division of Mental Health, Kenya Medical Research Institute, Nairobi, kenya; 21 Center for Global Mental Health Equity, Department of Psychiatry and Behavioral Health, George Washington University, Washington, DC, USA; 22 College of Education and Behavoural Studies, Arsi University, , Arsi Asela, Ethiopia; 23 Department of Psychiatry, School of Medicine, College of Health Sciences, Addis Ababa University, Addis Ababa, Ethiopia; 24 Deputy Chief Executive, Mental Health Authority, Accra, Ghana; 25 Mental Health Department, Institutional Care Division, Ghana Health Service, Accra, Ghana; 26 Kitui County, Ministry of Health, Nairobi, Kenya; 27 Federal Ministry of Health of Ethiopia, Addis Ababa, Ethiopia; 28 National Psychosis Unit, South London and Maudsley NHS Foundation Trust, London, UK; 29 Department of Psychological Medicine, King’s College London, London, UK; 30 Department of Public Health Sciences, Faculty of Life Sciences and Medicine, King’s College London, London, UK; 31 Centre for Global Mental Health and Centre for Implementation Science, Health Service and Population Research Department, Institute of Psychiatry, Psychology & Neuroscience, King’s College London, London, UK; 32 Department of Psychiatry and WHO Collaborating Centre in Mental Health Research and Capacity-Building, School of Medicine, College of Health Sciences, Addis Ababa University, Addis Ababa, Ethiopia

**Keywords:** community mental health, mental illness stigma, minority issues and cross-cultural psychiatry, psychosis, social inclusion

## Abstract

**Aim:**

HOPE (National Institute for Health and Care Research Global Health Research Group on Homelessness and Mental Health in Africa) aims to develop and evaluate interventions that address the unmet needs of people who are homeless and have severe mental illness (SMI) living in three African countries in ways that are rights-based, contextually grounded, scalable and sustainable.

**Methods:**

We will work in the capital city (Addis Ababa) in Ethiopia, a regional city (Tamale) in Ghana, and the capital city (Nairobi) and a rural county (Makueni) in Kenya to understand different approaches to intervention needed across varied settings.

We will be guided by the MRC/NIHR framework on complex interventions and implementation frameworks and emphasise co-production. Formative work will include synthesis of global evidence (systematic review, including grey literature, and a Delphi consensus exercise) on interventions and approaches to homelessness and SMI. We will map contexts; conduct focused ethnography to understand lived experiences of homelessness and SMI; carry out a cross-sectional survey of people who are homeless (n = 750 Ghana/Ethiopia; n = 350 Kenya) to estimate prevalence of SMI and identify prioritised needs; and conduct in-depth interviews and focus group discussions with key stakeholders to understand experiences, challenges and opportunities for intervention. This global and local evidence will feed into Theory of Change (ToC) workshops with stakeholders to establish agreement about valued primary outcomes, map pathways to impact and inform selection and implementation of interventions. Intervention packages to address prioritised needs will be co-produced, piloted and optimised for feasibility and acceptability using participatory action research. We will use rights-based approaches and focus on community-based care to ensure sustainability. Realist approaches will be employed to analyse how contextual variation affects mechanisms and outcomes to inform methods for a subsequent evaluation of larger scale implementation. Extensive capacity-strengthening activities will focus on equipping early career researchers and peer researchers. People with lived experience of SMI and policymakers are an integral part of the research team. Community engagement is supported by working closely with multisectoral Community Advisory Groups.

**Conclusions:**

HOPE will develop evidence to support action to respond to the needs and preferences of people experiencing homelessness and SMI in diverse settings in Africa. We are creating a new partnership of researchers, policymakers, community members and people with lived experience of SMI and homelessness to enable African-led solutions. Key outputs will include contextually relevant practice and policy guidance that supports achievement of inclusive development.

## Background

People with severe mental illness (SMI; comprising disabling psychoses and affective conditions) are over-represented in homeless populations globally. However, the situation is stark in low- and lower-middle-income countries (LLMICs), where an estimated 28–36 million people are homeless and have SMI (Chamie, 2017; Smartt *et al.*, 2019). In systematic reviews from high-income countries, the co-occurrence of SMI and homelessness is associated with numerous adverse outcomes, including premature mortality (Fazel *et al.*, 2014); infectious disease (chiefly tuberculosis, HIV and hepatitis B)(Beijer *et al.*, 2012); non-communicable diseases (Scott *et al.*, 2013); co-morbid alcohol and substance abuse; injuries and accidents (Mackelprang *et al.*, 2014); and suicide (Arnautovska *et al.*, 2014). Women and youth who are homeless are at particular risk of sexual assault and exploitation (Goodman *et al.*, 1995). SMI is a major risk factor for chronic homelessness (Fazel *et al.*, 2014).

Despite strong social capital and protective family structures in many LLMIC settings in Africa, caring for a person with SMI can overwhelm informal support networks, exacerbated by precarious household finances, stigma, diverse understandings about mental illness and limited mental healthcare provision (Read *et al.*, 2009). Harsh conditions on the streets can also trigger new onset of SMI. High-quality evidence from LLMICs is scarce, but most people who are homeless and have SMI have unmet basic needs for water, clothing and food, and untreated medical problems; 30–40% are affected by physical disabilities (Fekadu *et al.*, 2014; Singh *et al.*, 2016; Tripathi *et al.*, 2013). Beyond basic needs, there is evidence of high levels of unmet needs for social support, relationships and rehabilitation, and of exposure to unjust imprisonment, exploitation, physical and sexual abuse. Few (10%) have ever received mental healthcare, and almost none receive adequate ongoing care.

The evidence base for intervention in high-income countries takes housing as an essential aspect of service provision for people who have SMI and are homeless, particularly the Housing First model (Tsemberis, 2011), which improves a wide variety of health and social outcomes (Woodhall-Melnik and Dunn, 2016). Other evidence-based health interventions include tailored primary healthcare programmes, care coordination, assertive community mental health treatment and critical time interventions (Hwang and Burns, 2014). The applicabilityof such models to LLMICs may be limited, given their reliance on access to housing, specialist multidisciplinary workers and government-funded social welfare and healthcare services. Furthermore, factors causing or maintaining homelessness in people with SMI in LLMICs vary and demand different responses, for example, in relation to mental health stigma and discrimination, forced displacement and poverty. A decolonial approach to research is therefore required – generating evidence that is centred on lived experience and embedded in local contexts – to stimulate incremental, organic changes that will positively impact the lives of people who are homeless with SMI (Abimbola, 2021).

In our scoping review (Smartt *et al.*, 2019), we were unable to identify any rigorous evaluations of programmes for people who are homeless and have SMI in LLMICs. Most identified programmes are run by stand-alone, non-governmental organisations (NGOs), rarely integrated with government provision (Narasimhan *et al.*, 2019), thus limiting scalability and sustainability. Crucially, little attention is paid to social needs and the preferences of those with lived experience, who may not wish to return to their communities of origins, due to ‘push’ factors such as shame, a need to escape from conflict, broken relationships, exploitative work, or other forms of abuse and exclusion, or because of ‘pull’ factors related to perceived opportunities and freedoms in cities (Haile *et al.*, 2020).

There remains a crucial gap in evidence and global recommendations of care for people with SMI who are homeless. Any effective response will require coordination and close collaboration of specialist and community-based care across sectors, particularly when family supports are overwhelmed or absent, as well as the meaningful involvement of people with lived experience. This evidence gap indicates a pressing need for an Africa-led partnership of researchers and key stakeholders to conduct high-quality research in this area.

The aim of HOPE (National Institute for Health and Care Research Global Health Research Group on Homelessness and Mental Health in Africa) is to develop and evaluate approaches to addressing the unmet needs of people who are homeless and have SMI living in LLMICs in ways that are rights-based, contextually grounded, scalable and sustainable.

**Objectives**:

1. Establish a partnership of researchers, implementers, policymakers and people with lived experience.
2. Synthesise global evidence and obtain expert consensus on priority actions and approaches.
3. Work in distinctive settings in Ethiopia (capital city), Ghana (regional capital) and Kenya (capital city and rural county) to:
  - Identify the priority needs and valued outcomes of those with lived experience, and opportunities and challenges for intervention.
  - Integrate global and local evidence to select, co-produce and pilot interventions that target priority needs, in order to investigate acceptability and feasibility.
  - Evaluate the impact of interventions on the human rights and outcomes valued by people with lived experience and generate evidence on intervention costs, implementation processes and outcomes.
  - Pioneer the development of methods and ethical frameworks for future research and interventions.
4. Impact global policy and practice through translation of evidence into a ‘how-to’ guide to adapt and implement programmes in diverse LLMIC contexts.
5. Build sustainable capacity across partners that support South–South and South–North exchange of expertise and mutual learning, and develops individuals, teams, organisations and systems.

## Methods

**Figure 1.**
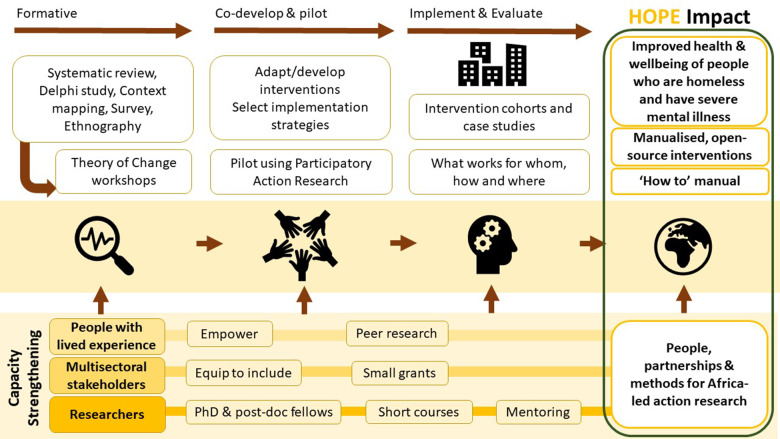
Overview of the HOPE project

See Fig. 1 for an overview of HOPE. HOPE is structured into work packages (WP): WP1 (project coordination), WP2 (formative phase), WP3 (intervention co-production/piloting), WP4 (implementation/evaluation), WP5 (capacity-strengthening) and WP6 (lived experience, community engagement and research uptake). In HOPE, we have prioritised the involvement of people with lived experience in all aspects of the research and delivery of interventions.

### Countries and settings

See Table 1 for characteristics of the countries and sites. These sites have been selected because (1) there is an evident need to improve support for people who are homeless and have SMI, (2) they are distinctive contexts in East and West Africa across low-income and lower-middle income countries; and (3) there are opportunities to build on existing programmes and political will. Homelessness has increased across the study countries, with accompanying challenges of poor health outcomes (Elsey *et al.*, 2019). In each study setting, social exclusion and stigmatisation of people who are homeless is common (UNICEF, MOLSA and Development Pathways, 2019), which intersects with stigma and discrimination experienced by persons with SMI (Forthal *et al.*, 2019; Mutiso *et al.*, 2019; Read *et al.*, 2009). Although all three countries are expanding community-based mental healthcare, these services are not tailored to the complex needs of people who are homeless and are inaccessible due to the reliance on families to bring a person to services and often meet the costs of treatment. Faith-based, civil society and NGOs contribute to crisis responses to basic needs for sub-groups of homeless populations but such efforts are fragmented (UNICEF, MOLSA and Development Pathways, 2019).

**Table 1.** Characteristics of countries and project sites in HOPE

	Ethiopia	Ghana	Kenya	
Country location	East Africa	West Africa	East Africa	
World Bank income category	Low-income	Lower-middle	Lower-middle	
Country population (World Bank Group 2023)	126 million	34.1 million	55.1 million	
Gross Domestic Product/capita (World Bank Group 2023)	$1294	$2238	$1950	
Number of mental health professionals/100,000 population	0.11 psychiatrists, 0.58 master’s level practitioners/psychiatric nurses, 0.045 clinical psychologists and 0.009 social workers (Federal Democratic Republic of Ethiopia Ministry of Health 2021)	0.07/100,000 (Roberts *et al.*, 2014)	0.19/100,000 (WHO 2014)	
Evidence on burden of homelessness and SMI	41% prevalence of SMI in sample of 217 people who were homeless in Addis Ababa, with extensive unmet needs (Fekadu *et al.*, 2014)	No prevalence estimates. Research has mostly focused on homeless youth in Accra (Asante *et al.*, 2014).	No data available	
Previous experience with interventions for people who are homeless and have SMI	Linked to expanding access to mental health care in a rural district in south-central Ethiopia (Smartt *et al.*, 2021)	In Tamale, community mental health workers assess and treat people who are homeless and have SMI within their catchment area.	In Nairobi, the CREATE initiative (Community Recovery Achieved Through Entrepreneurism) demonstrated how an integrated approach to social inclusion of people with SMI, including some who were homeless (Casey *et al.*, 2018) could support employment of people with SMI (MacDougall *et al.*, 2021)	
**HOPE project sites**				
	Ethiopia	Ghana	Kenya	
HOPE project sites	Addis Ababa sub-cities (Arada, Addis Ketema)	Tamale city	Nairobi county (Kamukunji, Starehe, Embakasi sub-counties)	Makueni county
Population of project site	4.4 million for Addis Ababa (United Nations Department of Economic and Social Affairs Population Division (2018) – with minor processing by Our World in Data 2018)	375,000 (Tamale Metropolitan Authority 2021)	4.4 million (United Nations Department of Economic and Social Affairs Population Division (2018) – with minor processing by Our World in Data 2018)	1 million (Ngugi 2013)
Characteristics of project site	These sub-cities have lower SES as well as concentrated numbers of people who are homeless compared with the other sub-cities in Addis Ababa. They also feature transport hubs as well as the main market in Addis Ketema. Overcrowding and poor quality of housing is a feature of both sub-cities, although new government-led housing projects have been constructed in the past 5−10 years.	Rapidly expanding regional capital situated 620 km from the capital; relatively lower socio-economic status in Ghana; situated on major internal and international transport routes; regional trading and agricultural centre	Nairobi occupies an area of about 700 km^2^ at the south-eastern end of Kenya’s agricultural heartland. Nairobi is facing challenges of rapid urbanisation, which is accompanied by informal settlements with poor quality of housing, as well as air and water pollution, water supply and sanitation.	250 km southeast of Nairobi, lower SES, with over 60% of the population living below the poverty line (Government of Makueni County 2018).
Mental health services	443 in-patient beds; hospital-based out-patient services; some integration in primary care; 1 residential rehabilitation facility	Hospital-based in-patient and out-patient services; community outreach by mental health nurses	Partially integrated mental health services at primary health care levels	Integrated mental health services at the primary healthcare level (Mutiso *et al.*, 2019)
Social care	Limited provision to meet basic needs from NGOs and religious institutions	NGOs and civil society organisations provide ad hoc support	Provision of social services is provided in a fragmented manner, either by faith-based organisations or NGO and other civil society organisations. There is a draft policy on street families that is yet to be finalised but no current services for people who are homeless.	No mental health or other public sector services for people who are homeless

### Conceptualising homelessness

Our starting point for defining homelessness is: spending the night unsheltered or in other places not intended for habitation. We will exclude people who spend their days on the streets, for example, to beg, but who have stable night-time accommodation. However, the concept of homelessness is complex and locally nuanced, so the target population will be operationalised for each study site in the formative phase.

### Theoretical framework

The research work in HOPE will follow the Medical Research Council/National Institute for Health and Care Research (MRC/NIHR) framework for developing and evaluating complex interventions (Skivington *et al.*, 2021). We will use ToC as a participatory tool to inform each aspect of the MRC/NIHR approach (De Silva *et al.*, 2014). Applying realist approaches, we will develop programme theories to inform evaluation. Unmet needs will be framed within a socio-ecological model that seeks to understand and propose interventions to address the individual, family, societal and political-level barriers to social inclusion.

### WP2: Formative phase

An extensive formative phase will comprise syntheses of global evidence and best practice alongside primary data collection in each setting: context mapping, ethnography and a cross-sectional study.

#### Systematic review

Building on our previous scoping review (Smartt *et al.*, 2019), we will conduct a systematic review, including grey literature, to identify interventions for people who are homeless with SMI in LLMICs (Smartt *et al.*, 2023). We will carry out a narrative synthesis of types of interventions, implementation strategies and evidence of impact.

#### Delphi consensus exercise

A Delphi consensus exercise will help identify global perspectives on best practices and priorities for interventions. We will invite people with experience of implementing relevant programmes in different regions in LLMICs, identified through the grey literature review and including French-, Spanish- and Portuguese-speaking implementers, and representatives of mental health service user associations, disability rights organisations and the World Health Organization (WHO). In the first round, participants identify interventions/components of programmes that they consider important. These will be consolidated and augmented by emerging findings from our review. In the second round, participants will rank each identified component/strategy based on importance and feasibility in LLMICs. Finally, the anonymised aggregated responses will be fed back to the participants, with further ranking to identify essential, desirable and optional elements of intervention programmes, as well as any ethical concerns.

#### Context mapping

We will conduct a documentary analysis of relevant policies, laws and plans relating to people who are homeless in our settings, inclusive of documents relating to public health, social care, legal decrees and other municipal documents. We will consult with community leaders and other key stakeholders in administrative positions, NGOs and religious and traditional healing sites. We will establish where people who are homeless and have SMI can be reached, and document the roles of different agencies and existing initiatives. Findings from the analysis will be synthesised using a matrix, informed by an implementation framework to map context (Pfadenhauer *et al.*, 2017), facilitating cross-country comparisons.

#### Primary data collection for formative studies

Primary data collection comprises a focused ethnography (Sangaramoorthy and Kroeger, 2020) and a cross-sectional study to understand experiences of homelessness and SMI, population burden, level of unmet needs, and sources of support, preferences, opportunities and potential barriers to intervention. We will carry out participant observation in locations where people are likely to sleep, visit or spend the day. Observations and linked interviews will focus on people who are homeless and have SMI, other members of the community, and their interactions. Interviews and focus group discussions will be conducted with key informants. The specific aims and methods are detailed in Table 2.

**Table 2.** Overview of methods for formative phase studies and pilot study

Study	Ethnography	Cross-sectional study	Pilot study
Aim(s)	To develop understanding of the lived experience of people who are homeless and have SMI, their unmet needs, priorities and preferences, and the contextual challenges and opportunities for intervention	To estimate the prevalence of SMI in homeless populations, evaluate validity of case identification by community health workers and investigate the profile of unmet needs, pathways into homelessness, experiences and family links.	To iteratively and collaboratively improve the intervention and implementation strategies, assess acceptability, feasibility and fidelity.
	To understand broader community responses to people who are homeless and have SMI, priority areas for intervention, experience of previous initiatives, potential challenges and how they may be overcome, as well as facilitators and resources to draw upon.		
Design	Focused ethnography, including context mapping, participant observation, and in-depth interviews and focus group discussions	Community-based survey based on methods used previously in Addis Ababa [Fekadu *et al.*, 2014].	Single-arm, feasibility study using Participatory Action Research
			The pilot evaluation framework will be informed by the ToC map.
Sites	Addis Ababa (Arada, Addis Ketema sub-cities); Tamale city; Nairobi county; Makueni county	Addis Ababa sub-cities (Arada, Addis Ketema); Tamale city; Nairobi county	Catchment area of a relevant facility, e.g. primary health care centre in Addis Ababa sub-cities (Arada, Addis Ketema); Tamale city; Nairobi county
Sample	Based on the mapping and consultation with stakeholders, we will purposively select locations for participant observation where people who are homeless and have SMI are likely to sleep or visit or spend the day. Linked interviews will be conducted with people with who are homeless (±SMI) and members of the public interacting with people who are homeless.	350−750 people aged 15 years or above who are homeless per site; 150 screened negative for SMI by health workers re-evaluated by mental health professional. For detailed sample size calculation, see Supplementary File 1.	10–15 people who are homeless and have SMI per site.
	30−35 in-depth interviews and 3−5 focus group discussions per site with purposively selected key informants, including people with lived experience, family members, social and health care providers, community leaders, religious and faith healers, policymakers, planners	Working through gatekeepers, each day we will demarcate a geographical area within the study site and start work early in the morning to identify all adults who are homeless at that location.	People involved in delivering interventions and those involved in the participatory action research (n = 6−8 per group per site, including people with lived experience).
		The ethnography will inform strategies to identify and engage with marginalised or disadvantaged subgroups of people who are homeless. Repeated visits to recruitment sites will seek to identify mobile and transient sub-populations.	
Procedures	Working through gatekeepers, the researchers will observe and speak informally with people such as market stall owners, food sellers, taxi drivers, as well as people who are homeless, noting aspects of the environment, observing interactions between persons who are homeless and members of the public, and asking observed people for clarifications and explanations.	Interviews will be carried out in private areas, by community nurses.	We will collect: (i) quantitative process indicators including training attendance, number of homeless people with SMI reached, engagement with interventions; (ii) observation checklist ratings of fidelity of intervention delivery and implementer competence; (iii) implementation log, supervision records and documentation of the PDSA cycle activity and emerging learning; and (iv) semi-structured interviews with intervention participants and implementers.
	Observations will be carried out at different times of day and in different locations over a period of 3–4 months. Observations will be documented in field notes and photographs.	Measures: (i) basic socio-demographic information, homelessness duration and experiences; (ii) presence of possible SMI focusing on behavioural manifestations; (iii) Camberwell Assessment of Need Short Appraisal Schedule (CANSAS Thornicroft *et al.*, 2006)^a^; (iv) Alcohol, Smoking and Substance Involvement Screening Test (ASSIST) [Humeniuk *et al.*, 2008]^b^; (v) suicidality; and (vi) structured interview with key informant if available.	In the pilot study, we will test out strategies to reduce attrition from services and from the project. These will depend on the site, and may include identifying contact mechanisms, e.g. through a peer, making regular contacts and provision of mobile phone top-up cards.
	All participants for in-depth interviews or focus group discussions will provide informed consent. Interviews will be conducted in a private place. Topic guides will be customised to respondent type. Each FGD will comprise 8–10 participants, seeking to ensure homogeneity with respect to power dynamics and hierarchies. In-depth interviews and FGDs will be audio-recorded, if permission is granted by the respondents; otherwise, detailed notes will be taken. Interviews/FGDs will be conducted primarily in local languages, transcribed verbatim in the original language and translated into English.	Mental health professionals will carry out a pragmatic diagnostic assessment based on the WHO Schedules for Clinical Assessment in Neuropsychiatry (SCAN)^a^ [McGuffin *et al.*, 1991] and items from the Psychosis Screening Questionnaire^a^ [Bebbington and Nayani 1995]. Those with a confirmed diagnosis of SMI (psychosis, affective disorder) according to ICD-11 criteria will undergo further assessment of duration of SMI, unmet needs using CANSAS to assess reliability with community nurse ratings and links with family and pathways into homelessness.	
Analysis	We will use an interpretative approach to analysis [Braun and Clarke 2019], focused on developing in-depth contextual understanding of the social experience of homelessness and SMI. We will analyse the interviews and FGDs using reflexive thematic analysis [Braun and Clarke 2019] and triangulate with the ethnographic field notes. Findings will be mapped onto an implementation research determinant framework (the Context and Implementation of Complex Interventions framework; CICI [47]) to identify potential micro, meso and macro level barriers and facilitators to programme success.	We will estimate prevalence of SMI and present descriptive data on unmet needs, substance use disorders and suicidality stratified by SMI status and gender. Profiles of unmet needs will be examined using latent class analysis (https://cran.r-project.org/web/packages/poLCA/). Multivariable modelling of association between latent classes of needs and SMI status, substance use disorders and recency of homelessness.	Process data summarised descriptively. Thematic analysis for qualitative data. Findings interpreted in relation to the ToC initial programme theories. Mapping findings using the CICI framework to identify determinants of implementation [Pfadenhauer *et al.*, 2017].

a Previously adapted for homeless populations in Ethiopia [Fekadu *et al.*, 2014].

b Previously adapted for Ethiopia and Kenya.

### Ethics

Key ethical challenges will now be highlighted: consent, responding to basic needs and safeguarding.

#### Consent to participate

In the ethnography, gatekeeper permission will be obtained. Aligned with accepted ethics of ethnographic practice, we will not seek formal consent from individuals who are being observed. The purpose of participant observation is to access an understanding of the day-to-day lives of people who are homeless and have SMI which might not be obtained via other methods, such as formal qualitative interviews. The process of obtaining informed consent can disrupt participants’ natural behaviours, actions and responses, including their relationship with the researcher-observer, risking the valuable insights ethnography might add. This was a point of contention in the HOPE team, with concerns raised by the Lived Experience Advisory Group (LEAG; below) about the dignity and autonomy of the person. To address these concerns, we will seek to build rapport with people who are homeless and have SMI over time through regular visits by the researchers and informal conversation, an approach used to engage homeless people with SMI in similar settings (Eaton *et al.*, 2015). This will facilitate respectful engagement with the person, allowing them to gain an understanding of the researcher’s role and develop trust. The researchers will be carefully trained and supervised to ensure they respect any desire not to be observed and cease observation if a person communicates discomfort (verbally or non-verbally). For in-depth interviews, informed consent will be obtained.

In the cross-sectional study, we will include people who lack mental capacity to provide informed consent as participants in the study with appropriate safeguards. We argue that the ethical principle of justice is upheld because the study enables us to obtain an understanding of the needs of the most vulnerable people who are homeless and have SMI and design interventions that best meet those needs. The United Nations Convention on the Rights of Persons with Disabilities implies a presumption of equal treatment with others who may wish to participate in research (i.e. having equal legal capacity), with the proviso that the prospective participant’s will and preference is always actively recognised and respected. We will not include anyone in the study if they indicate refusal or if there is any other indication that their will and preference would be not to participate. To identify evidence of the latter, we will seek to speak to a trusted person who is nominated by the person who is homeless and has SMI. That person could be anyone who credibly supports the individual. In the absence of such a person, a mental health/disability advocate will communicate with the person, seek to identify their will and preference and provide permission for the person’s participation. Mental capacity will be assessed by a mental health professional, using a standardised approach (Hanlon *et al.*, 2016). As a further safeguard for study participants who lack mental capacity, we will make systematic efforts to reassess capacity after 2 weeks, during which time we will support them to access mental health services. If the person has regained capacity, they will be invited to provide informed consent to participate and, if they decline, they will be withdrawn from the study.

#### Responding to basic needs

During the ethnography fieldwork, researchers may offer refreshments to people who are homeless during informal interactions as an act of reciprocity and following altruistic norms. For the cross-sectional survey, refreshments will be made available for all people who are homeless in the vicinity, regardless of study participation, so as not to provide undue incentives or coercion to participate. People with emergency medical needs will be supported to access care, working with local healthcare services and community health workers. When safeguarding needs surface, we will respond as detailed below. Participants will be informed about resources in the vicinity where they may access support, e.g. in relation to shelter, feeding programmes, local health services, and support for vulnerable women. For people with SMI, concerted efforts will be made to link them to mental health services (according to their preference). Involuntary mental healthcare will be utilised only in line with the country’s legislation (Ghana, Kenya) or, where no legislation exists (Ethiopia), if the person’s mental state is assessed as posing an imminent risk of harm to themselves or others. In the previous study from Ethiopia (Fekadu *et al.*, 2014), only 2 out of 89 people who were homeless and had SMI received involuntary psychiatric treatment.

#### Safeguarding

Standard operating procedures (see supplementary file 2) have been developed to support researcher responses to safeguarding concerns arising from (1) chaining, restraint or seclusion; (2) sexual abuse, exploitation or harassment; (3) physical abuse or serious physical health concerns; (4) suicidal behaviour; (5) violent or aggressive behaviour; (6) human rights abuses by professionals or community members; and (7) child trafficking or other child protection needs. Community advisory boards, including persons with lived experience, informed the development of localised protocols.

### WP 3: Intervention co-development and piloting

#### Participatory ToC workshops and intervention selection

We will conduct ToC workshops at the beginning and end of the formative phase. Each ToC workshop will last 0.5–1 day. Participants will include stakeholders identified through the formative work. Workshops will start by developing agreement on the desired long-term outcomes and impacts of the programme, then map out the interventions needed to achieve intermediate outcomes, identifying underlying assumptions and barriers, potential implementation strategies, required resources and inputs, and indicators of success. Global and country-specific evidence and experience arising from the formative phase findings will be integrated into the initial programme theory derived from the ToC workshops.

Interventions with evidence of effectiveness in low- or middle-income countries identified through our reviews and Delphi exercise will be examined for relevance to preferences, unmet needs and feasibility of adaptation (based on formative studies). We will identify where new intervention development is needed (Box 1). This process will initially be undertaken within each country by a small (n = 5–8) working group comprising people with lived experience of SMI, potential implementers and the HOPE country project teams. HOPE consortium members will then review the proposed interventions/approaches and suggest further ways to tailor the interventions to the local context. We will give particular focus to rights-based approaches, potential scalability and sustainability, and identifying the role of peer support.

#### Co-development of interventions and implementation strategies

Working groups of people with lived experience, implementers and the research team will meet several times to co-produce the interventions, implementation strategies and fidelity checklists. We will describe the interventions according to recommended guidance (Hoffmann *et al.*, 2014) and catalogue the implementation strategies according to existing taxonomies (Powell *et al.*, 2015) to enhance testing in new settings. The FRAME (Framework for Reporting Adaptations and Modifications Expanded) implementation tool (Wiltsey Stirman *et al.*, 2019) will be used to document intervention adaptations and their justification. We anticipate that the resulting intervention packages for the three study settings will have elements that are similar, although addressed in differing ways depending on the context (e.g. accessing physical and mental healthcare, addressing basic needs, common elements of needs assessment and planning, peer support, individual engagement, addressing social exclusion) as well as elements that may be site-specific (e.g. addressing substance misuse, family interventions, housing). Candidate personnel for delivering and supervising the intervention/s will be existing human resources, such as community health workers, peers (people with lived experience), the NGO sector, social workers, primary healthcare staff and psychiatric nurses.

#### Realist methods

We will conduct a realist synthesis to inform our understanding of what works, for whom, in what circumstances and why (Wong *et al.*, 2017). Drawing on cross-country analyses of findings from the formative phase, we will map our initial ToC-based programme theories onto mid-range programme theories and, in collaboration with key stakeholders, develop hypotheses about the important ways that interventions can bring about change in different contexts, i.e. ‘context–mechanism–outcome configurations’. These will then be explored in the pilot phase and used to develop a realist evaluation framework for larger-scale implementation.

Box 1. **Candidate interventions for adaptation**

HOPE co-investigators have been involved in the development, implementation and evaluation of several interventions for people with SMI in the study countries which have potential relevance to homeless populations. Materials (manuals, training materials) and implementation strategies (e.g. for competency-based training and supervision (Laura Asher et al. 2021)) are available for the following: community-based rehabilitation for people with SMI delivered by lay workers (Asher et al. 2022; Asher et al. 2018), social contact-based stigma reduction interventions (Kohrt et al. 2021), awareness-raising and community outreach for people who are homeless and have SMI, engagement with traditional and religious healers (L. Asher et al. 2021; Yaro et al. 2020), task-shared models of integrated mental health care in primary health care (Hanlon et al. 2022; Hanlon et al. 2019; Mutiso *et al.*, 2019), brief psychological interventions (Bitew et al. 2021), brief interventions for substance use disorders (Clair et al. 2019; Hailemariam et al. 2016; Harder et al. 2020) and gender-based violence (Keynejad et al. 2020), family interventions (Asher et al. 2018; Casey et al. 2018), livelihoods interventions (BasicNeeds-Ghana; Lund et al. 2013), addressing basic needs (BasicNeeds-Ghana), self-help groups and peer support (Cohen et al. 2012; Puschner et al. 2019). In the ongoing SCOPE project (Ethiopia), we are developing a toolkit for early identification of people who are homeless and have SMI (Hanlon et al. 2023).

#### Pilot study

The resulting intervention package will be piloted in each country in a circumscribed geographical area linked to a primary healthcare centre or other relevant facility. We will conduct a single-arm feasibility study over 3 months, focusing on initial assessment and care planning, engagement and early interventions (see Table 2). Based on the ToC and realist synthesis, we will identify process indicators (spanning quantitative and qualitative data) for each intermediate outcome and explore potential mechanisms through which context influences outcomes. We will examine the feasibility and appropriateness of using routinely collected service indicators and identify where project data will be needed as the basis for monitoring and evaluation, and quality improvement. We will initially run training for a small group of implementers. Regular meetings (every 1–2 weeks) with implementers and people with lived experience, supported by the project team, will use plan-do-study-act (PDSA) cycles (Taylor *et al.*, 2014) to identify and address emerging challenges, allowing iterative improvement of the intervention and approaches. The structures and supports that underpin this co-production approach are the focus of WP5 and WP6.

#### Costing interventions

Proformas will be designed and tested to facilitate key decisions about our costing approach; for example, determining which intervention components can be reliably estimated at an individual (bottom-up) level and which will require an aggregate (top-down) approach. In semi-structured interviews with implementers, we will gain views on the extent to which additional tasks related to the intervention replace or add to existing activities, to identify possible resource allocation for services or organisational implications that need to be scrutinised in the larger-scale implementation and evaluation study. A service use data collection form will be adapted and piloted.

### WP4: Implementation and evaluation

Based on pilot study findings, the country working groups will finalise interventions and implementation strategies. We will then carry out phased implementation and theory-driven evaluation of the intervention package at a larger scale in the formative study sites. A study protocol will be reported separately.

### WP5: Capacity-strengthening

We are not aware of a partnership focused on addressing needs of people who are homeless and have SMI in Africa. Tackling this problem mandates use of research methodologies that have not been widely used in global mental health, including ethnography and participatory action research. To be successful, the partnership also needs to expand research capabilities to peer researchers and implementers, build researcher capacity for rights-based work and strengthen capacity for multi-sectoral working that is respectful and inclusive. We will deliver a programme of capacity-strengthening that builds on identified capabilities, is tailored to the different needs across countries and institutions, links directly to the work in HOPE, is designed for sustainability and is evaluated for impact (Hanlon *et al.*, 2018) (see Table 3). Guided by the ESSENCE framework (TDR/World Health Organization, 2016), our capacity-strengthening efforts will be evaluated for impact by collecting the following data: process indicators (participants in training courses and webinars: gender, age and background of participants; uptake and completion of training), satisfaction (anonymous online survey), impact (publications, percentage of first authors from LMICs, with lived experience and/or who are female, grant applications and conference presentations).

**Table 3.** HOPE capacity-strengthening activities

Target groups and activities			
Group	Numbers and location	Tailored activities	Cross-cutting activities
PhD and post-doctoral fellowships	2 PhDs (Ethiopia, Ghana)	Registered in public sector universities; External supervisors;	Journal clubs
	3 Post-doctoral fellows (Ethiopia, Ghana, Kenya)	Mentorship	Online mentoring of context mapping and ethnography
Other early and mid-career researchers	5 co-investigators within Ethiopia, Ghana and Kenya	Mentorship	Systematic review collaboration
Research team members	5 research coordinators and assistants employed on HOPE		Short courses and webinars
People with lived experience	6 peer researchers (2 per country)	Stipend for 50% time Small grant 1:1 supervision and support	NIHR online learning on Community Engagement and Involvement in Global Health Research (National Institute for Health and Care Research in collaboration with Mesh Community Engagement Network 2024)
**Short courses and webinars**			
Completed			
Ethnography (3 days: Addis Ababa; March 2023)			
Structural equation modelling (2 days: Addis Ababa, March 2023)			
Participatory action research (2 days: Addis Ababa, March 2023)			
Theory of Change (bespoke online course run by Dr Erica Breuer; 5 × 2 hour training sessions, 2 supervision sessions)			
Planned in 2024			
Peer support training for lived experience representatives			
PhotoVoice training as a tool for empowerment of people with lived experience			
Tailored research methods for lived experience group			
Analysis and write-up of ethnographic research, including application of social theory			
Webinars on complex intervention development/adaptation, piloting and evaluation* (open)			
Webinars on programmes for people who are homeless and have severe mental illness (open)			
Writing workshops for academic paper-writing			

### WP6: Lived experience, community engagement and research uptake

Mental health service user and advocacy organisations, and representatives from Ministries of Health in each partner country, contributed to the development of the HOPE proposal and were named collaborators on the grant application, in recognition of the fundamental importance of their active engagement in HOPE. A LEAG has been established and meets monthly to provide input into evolving study protocols and standard operating procedures, especially in relation to ethically sensitive aspects of the study. The LEAG feeds back to the HOPE steering committee and investigator group monthly and has directly influenced methods for ethnography and the survey. We are seeking to expand the LEAG to include people who have lived experience of homelessness as well as SMI, supported by empowerment methods such as PhotoVoice (Rai *et al.*, 2023).

Policymaker collaborators and World Health Organization representatives also join steering committee meetings on a quarterly basis and attend the annual meeting, with interim engagement within countries. In these meetings, priority is given to understanding the country level and global policy context and opportunities for HOPE to achieve impact, as well as keeping colleagues informed about, and engaged with, developing plans and emerging findings. Local community and stakeholder engagement is achieved through multisectoral community advisory boards and national level country management groups. Ongoing engagement and targeted messaging are key aspects of our research uptake strategy, focused on achieving high levels of ownership and the potential for sustainable impacts. We will monitor policy uptake of HOPE outputs.

## Discussion

### Potential challenges

The HOPE programme is ambitious and faces important structural barriers to its success. These include stigma and the limited availability of social welfare provision and services tailored to the needs of people with SMI who are homeless. For sustainability we are focusing on mobilising existing resources and ensuring fair access, but we will also use findings to advocate for greater prioritisation of this group in resource allocation.

### Practice implications

Our inclusive approach to partnership combined with our focus on the preferences and priorities of people with lived experience of SMI and/or homelessness marks an important change to existing practice. The resulting intervention packages will seek to coordinate services around individuals rather than vice versa, with efforts to avoid vertical programming. The importance of multi- and inter-sectoral programming to support recovery of people with SMI has been highlighted (van Rensburg and Brooke-Sumner, 2023). Lessons learned from HOPE will have relevance for other populations of people with SMI who have complex needs and WHO’s renewed efforts to promote de-institutionalisation of mental health care (WHO, 2022).

### Policy implications

The HOPE NIHR Global Health Research Group will speak directly to the United Nations Sustainable Development Goal imperative that ‘no one should be left behind’ in development efforts (Patel *et al.*, 2018), seeking to address the systematic exclusion of people who are homeless and have SMI from key services and societal opportunities. While the imperative for social inclusion of people who are homeless, including those with SMI, is emphasised by the United Nations (United Nations Secretary General, 2023), governments and implementing organisations are hampered by a lack of compelling and fit-for-purpose evidence; and there is a particular evidence gap in LLMICs. In HOPE, we will generate evidence on rights-based, contextually relevant, effective and scalable interventions for people who are homeless and have SMI.

## Supporting information

Hanlon et al. supplementary material 1 Hanlon et al. supplementary material

Hanlon et al. supplementary material 2 Hanlon et al. supplementary material

## Data Availability

The paper describes planned work and does not make use of any data.

## Supplementary material

The supplementary material for this article can be found at https://doi.org/10.1017/S2045796025000186.
