# Supplementary material for: Improving outcomes for people who are homeless and have severe mental illness in Ethiopia, Ghana and Kenya: overview of the HOPE programme
Source: Epidemiol Psychiatr Sci. 2025 Apr 21;34:e26. doi: 10.1017/S2045796025000186 (PMC7617600; doi:10.1017/S2045796025000186)
Supplement: Hanlon et al. supplementary material 1 — Hanlon et al. supplementary material [file S2045796025000186sup001.docx]

Supplementary File 1

*Sample size calculation for cross-sectional study*

Assuming a prevalence of SMI up to 50% [1], for precision of =/-5% around the prevalence estimate, a sample of 400 people who are homeless is required per site (using a single-proportion formula). This sample size is feasible in all sites. To mitigate diagnostic accuracy issues in the field, we need to recruit more than 400 study participants. Furthermore, to look for distinct profiles of unmet needs, latent class analysis (see below) typically requires large sample sizes to ensure model convergence and proper estimation (n = 400-1000 according to empirical and simulation studies)[2]. We anticipate that in Addis Ababa, Tamale and Nairobi it will be feasible to recruit n=750 per site.

To evaluate the diagnostic accuracy (sensitivity and specificity) of community screening for people with psychosis. We assume that mental health professional assessment with SCAN (the semi-structured diagnostic assessment) is the gold standard. If prevalence of severe mental illness in people experiencing homelessness = 0.55, and

- expected sensitivity = 0.65 (this is the proportion of true positives correctly identified by community nurses)
- expected specificity = 0.95 (this is the proportion of true negatives correctly identified by community nurses)

With an expected dropout rate = 0.20, a sample of 199 would be required to estimate with 5% precision

- empirical sensitivity values that range from 0.55 - 0.75
- empirical specificity values that range from 0.85 - 1.00

Given 200 individuals who screened positive and 150 who screened negative, the target sample size for stage 2 screening is 350. This thus meets the stipulated sample size requirements. Sample size calculated using [https://wnarifin.github.io/ssc/sssnsp.html](https://eur03.safelinks.protection.outlook.com/?url=https%3A%2F%2Fwnarifin.github.io%2Fssc%2Fsssnsp.html&data=05%7C01%7Ccharlotte.hanlon%40kcl.ac.uk%7C0fe807b8373e4f61292108db61184a35%7C8370cf1416f34c16b83c724071654356%7C0%7C0%7C638210529177035294%7CUnknown%7CTWFpbGZsb3d8eyJWIjoiMC4wLjAwMDAiLCJQIjoiV2luMzIiLCJBTiI6Ik1haWwiLCJXVCI6Mn0%3D%7C3000%7C%7C%7C&sdata=z%2FJovISwWkG5xCA3mPTA1gWTAxmzZqP821YrNb1MOwA%3D&reserved=0)

1. Smartt C, Prince M, Frissa S, Eaton J, Fekadu A, Hanlon C. Homelessness and severe mental illness in low- and middle-income countries: scoping review. BJPsych open. 2019;5(4):e57. Epub 2019/09/19. doi: 10.1192/bjo.2019.32. PubMed PMID: 31530300; PubMed Central PMCID: PMCPMC6611071.

2. Dziak JJ, Lanza ST, Tan X. Effect Size, Statistical Power, and Sample Size Requirements for the Bootstrap Likelihood Ratio Test in Latent Class Analysis. Structural Equation Modeling: A Multidisciplinary Journal. 2014;21(4):534-52. doi: 10.1080/10705511.2014.919819.
