# Supplementary material for: Improving outcomes for people who are homeless and have severe mental illness in Ethiopia, Ghana and Kenya: overview of the HOPE programme
Source: Epidemiol Psychiatr Sci. Author manuscript; Available in PMC 2025 Apr 24. (PMC7617600; doi:10.1017/S2045796025000186)
Supplement: Supplementary file 1 [file EMS204115-supplement-Supplementary_Materials.pdf]

Supplementary File 1

Sample size calculation for cross-sectional study

Assuming a prevalence of SMI up to 50% [1], for precision of =/-5% around the prevalence estimate, a sample of 400 people who are homeless is required per site (using a single-proportion formula). This sample size is feasible in all sites. To mitigate diagnostic accuracy issues in the field, we need to recruit more than 400 study participants. Furthermore, to look for distinct profiles of unmet needs, latent class analysis (see below) typically requires large sample sizes to ensure model convergence and proper estimation (n = 400-1000 according to empirical and simulation studies)[2]. We anticipate that in Addis Ababa, Tamale and Nairobi it will be feasible to recruit n=750 per site.

To evaluate the diagnostic accuracy (sensitivity and specificity) of community screening for people with psychosis. We assume that mental health professional assessment with SCAN (the semi-structured diagnostic assessment) is the gold standard. If prevalence of severe mental illness in people experiencing homelessness = 0.55, and

- expected sensitivity = 0.65 (this is the proportion of true positives correctly identified by community nurses)
- expected specificity = 0.95 (this is the proportion of true negatives correctly identified by community nurses)

With an expected dropout rate = 0.20, a sample of 199 would be required to estimate with 5% precision

- empirical sensitivity values that range from 0.55 - 0.75
- empirical specificity values that range from 0.85 - 1.00

Given 200 individuals who screened positive and 150 who screened negative, the target sample size for stage 2 screening is 350. This thus meets the stipulated sample size requirements. Sample size calculated using https://wnarifin.github.io/ssc/sssnsp.html

1. Smartt C, Prince M, Frissa S, Eaton J, Fekadu A, Hanlon C. Homelessness and severe mental illness in low- and middle-income countries: scoping review. BJPsych open. 2019;5(4):e57. Epub 2019/09/19. doi: 10.1192/bjo.2019.32. PubMed PMID: 31530300; PubMed Central PMCID: PMCPMC6611071.
2. Dziak JJ, Lanza ST, Tan X. Effect Size, Statistical Power, and Sample Size Requirements for the Bootstrap Likelihood Ratio Test in Latent Class Analysis. Structural Equation Modeling: A Multidisciplinary Journal. 2014;21(4):534-52. doi: 10.1080/10705511.2014.919819.

NIHR Global Health Research Group on Homelessness and Mental Health in Africa

Standardised operating procedures (SOPs) for safeguarding people who are homeless, including those with mental health conditions, in HOPE-Ethiopia

1 CONTENTS

2 Introduction 2
3 Purpose 2
4 Who is this SOP for? 2
5 Responsibility 3
6 Abbreviations 3
7 SOPs for protecting against harms due to the HOPE project 4
7.1 Potential harms due to the HOPE project 4
7.2 Protecting against, and responding to, harms from actions of a project worker 4
7.3 Protections for involvement of people with MHCs who lack decision-making capacity 5
7.4 Protecting against increasing mental health-related stigma and exclusion 9
8 SOPs for safeguarding concerns identified by HOPE project 10
8.1 Definitions of potential harms 10
8.2 Identification of harms 12
8.3 Actions to be taken upon identification of safeguarding concerns 12
8.3.1 Chaining, restraint or seclusion in public settings 12
8.3.2 Sexual abuse, exploitation or harassment 13
8.3.3 Physical abuse or severe ill-health 14
8.3.4 Suicidal behaviour or self-harm 15
8.3.5 Violent or aggressive behaviour 15
8.3.6 Coercive treatment, restraint or abuse on the premises of facilities or healing sites 17
8.3.7 People Trafficking or involvement in Drug Dealing or Criminal Activities 17
8.3.8 Neglect of children living on the street 17
9 Appendix: Flow charts for responses to safeguarding concerns 18

2 INTRODUCTION

This SOP builds on the CDT-Africa (Centre for Innovative Drug Development and Therapeutic Trials for Africa, Addis Ababa University) Safeguarding Policy. In this SOP we focus on specific procedures that are relevant to safeguarding within the HOPE project in Ethiopia.

In HOPE we will be working with people who are homeless, including those who have severe mental health conditions (MHCs). We will have contact with these individuals while on the streets and at the places where they seek help, including religious institutions, healing sites, health facilities, social care facilities and non-governmental organisations. People who are homeless are at increased risk of several types of harms and are considered a vulnerable group because of social exclusion and poverty. Those who are homeless and additionally have severe MHCs are at greater risk of harms.

In the HOPE project we have a responsibility to protect people who are homeless, with or without a severe MHC, from being exposed to harm during contacts with our project staff. We also have a responsibility to respond to any harms or safeguarding concerns that we identify in a timely and robust manner.

This Standardised Operating Procedure is based on previous approaches used when working with people with severe MHCs in the community in Ethiopia. Those ways of working were developed in collaboration with multi-sectoral Community Advisory Boards and members of the Mental Health Service User Association of Ethiopia. This draft SOP will be finalised with input from the HOPE Lived Experience Advisory Group and the Community Advisory Board in Ethiopia. We will include safeguarding as a standing item on the agenda of the twice-yearly Community Advisory Board meetings.

3 PURPOSE

This standard operating procedure (SOP) describes the process of avoiding, detecting and responding to harms and safeguarding concerns related to people who are homeless, including those who have severe MHCs, in the HOPE project.

4 WHO IS THIS SOP FOR?

This SOP applies to the following people:

- all HOPE investigators
- HOPE research staff (including the project manager, research co-ordinators, research assistants, field supervisors, peer researchers, clinicians involved in research assessments or interventions, data collectors)
- students linked to HOPE
- health extension workers, health extension worker supervisors, health workers in facilities and other professionals and community members (‘community supporters’) who are

involved in the identification, referral and interventions for people who are homeless, with or without severe MHCs, as part of HOPE project activities.
- community advisory board members

5 RESPONSIBILITY

- Everyone is responsible for working in a way that **protects people** who are homeless (with or without severe MHCs) against harms, conducting themselves in a way that **respects their rights**, and **reporting any concerns** about the behaviour of others promptly.
- Community supporters and community-based professionals (e.g., health extension workers) are responsible for detecting harms and safeguarding concerns, taking any necessary immediate measures and linking with the HOPE team for further actions.
- HOPE research staff working in the field are responsible for detecting and reporting harms and taking the appropriate measures, including when harms may have been perpetrated by other project staff members (whistle-blowing).
- A designated mental health focal person will support field staff in an immediate response to safeguarding concerns related to mental health, facilitate engagement with mental health care and following up to make sure that harms have been addressed to best of our ability.
- The project co-ordinator is responsible for co-ordinating a prompt response to safeguarding concerns, working with members of the research team to document any harms, concerns and actions taken (including for Serious Adverse Event reporting), and for promptly informing and involving the mental health focal person and Principal Investigators.
- Community advisory board members will promptly report any potential safeguarding concerns that they become aware of to the HOPE project team or safeguarding focal person at CDT-Africa and work closely with the HOPE team in addressing safeguarding concerns, depending on their specific area of expertise.

6 ABBREVIATIONS

HEW: Health Extension Worker

HOPE: Project on Homelessness and Mental Health in Africa

MHC: Mental Health Condition

PI: Principal Investigator

SAE: Serious Adverse Event

SOP: Standard Operating Procedure

7 SOPs FOR PROTECTING AGAINST HARMS DUE TO THE HOPE PROJECT

7.1 POTENTIAL HARMS DUE TO THE HOPE PROJECT

Harms due to the project may occur through:

(1) The actions of project staff that exploit the vulnerable status of a person who is homeless, including those with a severe MHC.
(2) Over-riding autonomy by inclusion of a person who is homeless and lacks decision-making capacity within the project.
(3) Increased stigma against people who are homeless and have a MHC.

7.2 PROTECTING AGAINST, AND RESPONDING TO, HARMS FROM ACTIONS OF A PROJECT WORKER

- All project staff employed on HOPE, students conducting projects on HOPE and HOPE investigators directly engaged in field work will first complete police checks.
- Staff, students and investigators will be trained in the Safeguarding Policy produced by CDT-Africa, Addis Ababa University and the specific HOPE safeguarding procedures.
- The safeguarding focal person for the HOPE project is the Project Co-ordinator. There is also a focal person for safeguarding in CDT-Africa.
- No person who is homeless, with or without an MHC, will be interviewed by a data collector or researcher in a private space on their own. If the person being interviewed is a woman, there will always be a woman present.
- Safeguarding concerns about a member of project staff, student or investigator can be reported by study participants to the ethics committee or using contact details on the participant information sheet.
- While conducting the community survey, the HOPE team will revisit recruitment sites 1-2 weeks after the initial assessment, giving people an opportunity to express any concerns directly.
- The community advisory board (CAB) will be an additional mechanism through which the project may hear about any concerns about project staff, investigators or students. The CAB should report any concerns directly to the HOPE safeguarding focal person or the CDT-Africa safeguarding focal person.
- If a HOPE project staff member, investigator or student observes concerning behaviour in another project staff member, investigator or student, they should immediately report this to the HOPE-Ethiopia PIs (Professor Abebaw Fekadu or Dr Ruth Tsigebrhan) or to the overall HOPE PIs (Professor Charlotte Hanlon or Professor Atalay Alem) or to the Safeguarding focal person for HOPE project or the CDT-Africa Safeguarding focal person. Who they report to directly may depend on who is involved in the safeguarding concern.
- The CDT-Africa Safeguarding Focal person should proactively engage with HOPE staff to provide opportunities for any concerns about project-linked people.

- If a safeguarding concern against a project staff member is reported to a PI, the PI is responsible for ensuring that the Safeguarding focal person at CDT-Africa is also informed. Following reporting, the CDT-Africa Safeguarding Policy guidance will then be followed with close input from the HOPE PIs. This includes considerations about whether the accused staff member needs to be suspended from field work, whether the police need to be involved and how the person who has been the victim of harms should be supported.

7.3 Protections for involvement of people with MHCs who lack decision-making capacity

In the HOPE project, people with severe MHCs who lack decision-making capacity to provide informed consent to participation in the study will be included in some aspects of the work, as approved by the Addis Ababa University College of Health Sciences Institutional Review Board (Ref 035/23/CDT; Date 19th April 2023) and King's College London Research Ethics Committee (HR/DP-22/23-34762; Date: 20th March 2023). In the formative phase, this is relevant to the cross-sectional survey.

The justification for involvement of people who are homeless and have a severe MHC but lack decision-making capacity is the ethical principle of justice, as follows:

A. Inclusion of a person who lacks capacity to consent is justified on the following basis: Inclusion will only occur if the impairment of capacity is due to a mental health condition (confirmed by a mental health professional).
B. Research of equal effectiveness could not be carried out if confined to participants with capacity. In the previous survey of people who are homeless conducted by our team in Ethiopia, 28/89 people who were homeless and had a severe MHC lacked capacity to consent. Exclusion of this group (31% of the target population) would substantially undermine the effectiveness of the research because the purpose is to understand unmet needs to develop interventions. Exclusion of those who are most unwell would mean that those with greatest need would be least likely to benefit from future interventions.
C. The research has potential to benefit the participant directly because we will arrange linkage to support services or clinical care as needed and in line with local laws, policies and guidance. The research will also provide knowledge of the unmet needs of people who are homeless and have severe MHC to inform the development of interventions that will be implemented and evaluated in the same setting during the HOPE project.

To minimise harms from over-riding individual autonomy, the assessments have been designed to be minimally burdensome (abbreviated clinical assessment, restricting self-report to the essential information), associated with minimal risk as there will be no invasive procedures (only observation or self-report questionnaires), and will not interfere significantly with privacy (assessments will take place in a private location, with chaperone as needed) or freedom (the person is free to walk away and any expression of refusal to participate will lead to their exclusion from the project).

Procedures and protections for involvement of participants who lack capacity to consent to participation are summarised in Flowcharts 1 and 2.

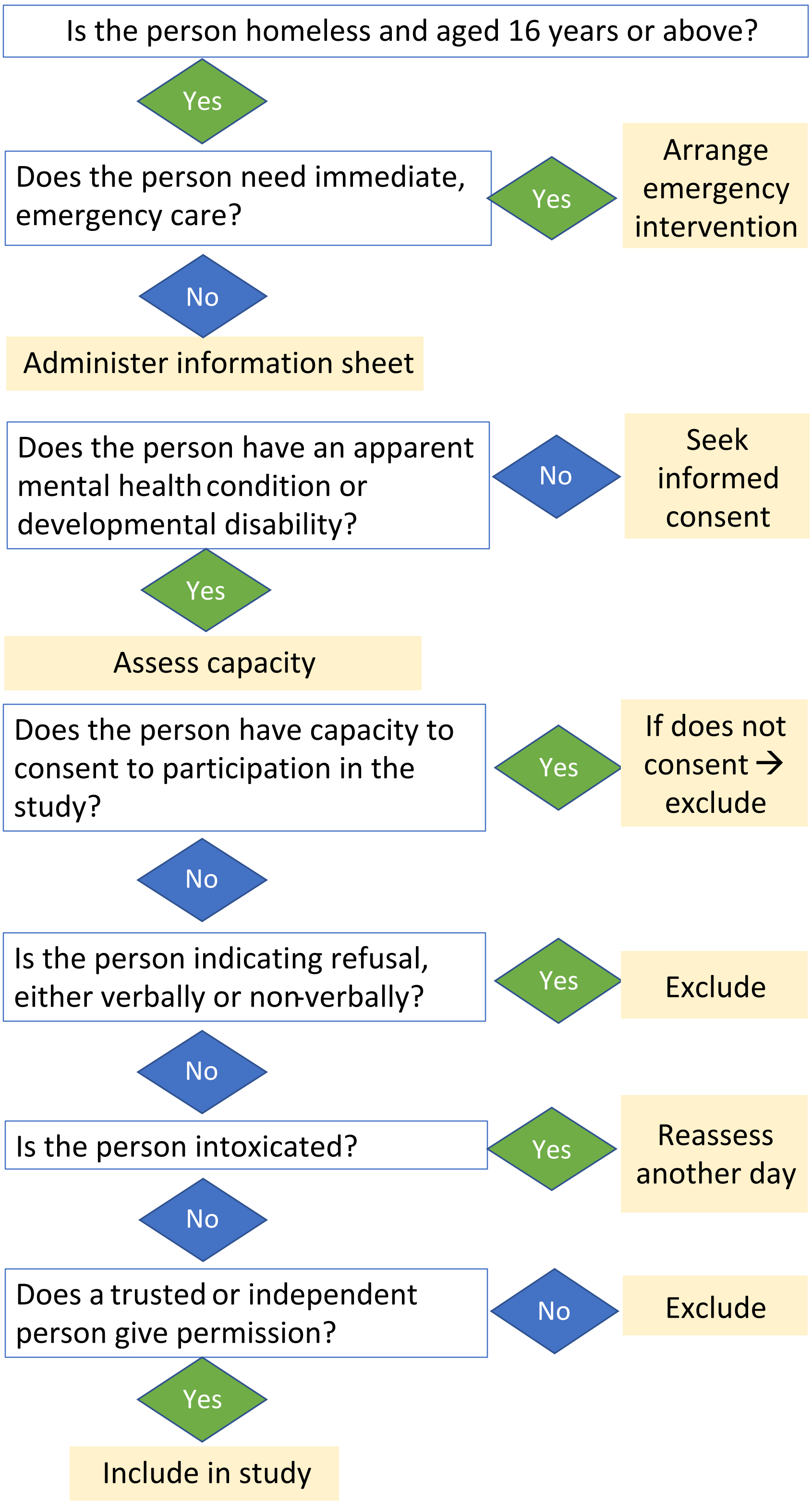

Figure 1: Overview of procedures for recruitment of a person with severe MHC who lack capacity to consent – Part 1

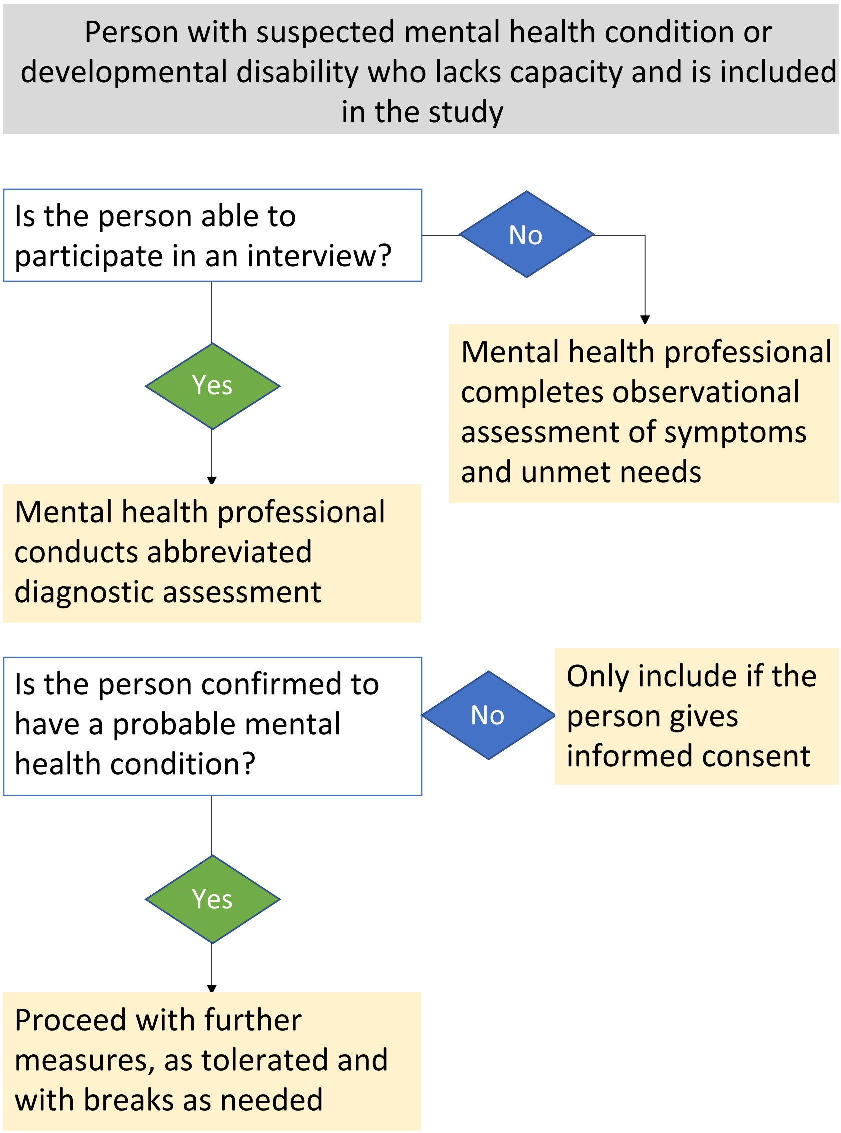

AFTER ASSESSMENT IS COMPLETED

- **Arrange for linkage to support services or clinical care as needed and in line with local laws, policies and guidance.**
- **Seek to follow-up after 1-2 weeks to re-assess capacity and willingness to participate in the study, and completion of self-report questionnaires if possible**

Figure 2: Overview of procedures for recruitment of a person with severe MHC who lack capacity to consent – Part 2

Key points are elaborated below:

1. **Capacity to consent:** Assessment of capacity to consent will be undertaken by trained mental health professionals using an approach used previously in Ethiopia[1] and requiring documentation of the key components of decision-making capacity: ability to understand information, retain it, weigh up the information and communicate a decision.
2. **Refusal:** No person communicating refusal or resistance to involvement in the study will be included.
3. **Maximising capacity to consent:** Through careful training of data collectors, we will seek to maximise an individual's capacity to consent. Efforts will be made to explain the points included within the information sheet and allowing sufficient time for the person to ask questions.
4. **Trusted or independent individual to provide permission:**
    a. We will seek to ask the person who lacks capacity if there is a trusted individual that they would like to nominate who knows their wishes.
    b. If this is not possible, we will seek to identify whether there is an individual who is supporting the person who lacks capacity. This could be a member of a local community-based organisation or member of the public. This individual should not be a law enforcement professional or a health professional who has a clinician-patient relationship with the person. We will seek to understand how well the 'supporter' knows the person who lacks capacity and will only involve consultees who appear to have a good understanding of the person and their preferences. If there have only been a few brief contacts, they would not be considered as a consultee.
    c. If such a person is not available, an independent consultee would consider the individual's circumstances and determine whether there is any evidence that the person does not want to be involved in the study and seek to identify what the person's decision would have been based on any available information, including discussion with the person. That independent consultee will be a person from the mental health service user association collaborating with HOPE or another disability rights organisation. They will be paid for their time but not in relation to the number of people recruited.
    d. Many persons who are homeless and who have severe MHCs have lost contact with their family and some may have difficult or abusive relationships with their family (sometimes the reason that they have become homeless). We will not, therefore, seek to contact family members about participation in the study unless the person themselves wishes this and can provide their contact details.
    e. Our approach to identifying an individual to provide permission will be in keeping with the UN Convention on the Rights of Persons with Disability principles of (1) 'Will and preference'; i.e. that any nominated person is expected to arrive at a conclusion about what the person would want based on the communication they can have with them (if independent) and their past knowledge and relationship with them if a known person; and (2) 'Supportive decision-making'; i.e. that the nominated person is not seeking to arrive at just a common-sense or even 'best interest' conclusion,

[1]Hanlon C, Medhin G, Dewey ME, et al. Efficacy and cost-effectiveness of task-shared care for people with severe mental disorders in Ethiopia (TaSCS): a single-blind, randomised, controlled, phase 3 non-inferiority trial. *Lancet Psychiatry* 2022;9(1):59-71. doi: 10.1016/s2215-0366(21)00384-9

but to know what the person's decision would be so that the person's preference is considered, whether or not they are deemed to have full capacity.

5. **Re-assessment of capacity and study withdrawal:** The person will be free to withdraw at any time. We will seek to re-assess capacity to consent and to provide opportunities for people who are included in the study to withdraw by re-visiting the recruitment area 1-2 weeks after recruitment.

For all other aspects of the HOPE formative work where consent is being obtained from a person with severe MHC, we will assess capacity at the point of recruitment and again before commencing the interview or other research activity. If during the course of the activity/interview it appears that the person does in fact not have capacity or is unable to understand the purpose of the interview/activity then we will stop the interview/activity and we will not use the data collected to that point. If the participant may regain capacity within the timescale of data collection, then we will invite them again to participate and re-assess capacity to provide informed consent. For the observational part of the ethnography, we will seek permission from gatekeepers, including police and city administration, and not from individuals.

7.4 Protecting against increasing mental health-related stigma and exclusion

We will seek to minimise the risk that the project could inadvertently increase stigma against people who are homeless and have a severe MHC as follows:

1. Training of all study investigators, staff and linked students on maintaining the dignity and human rights of people who are homeless, including those who also have a severe MHC.
2. Adequate supervision of project-linked people working in the field to ensure that their interactions with people who are homeless and have severe MHCs are respectful.
3. Ensuring privacy when interviews and assessments are being undertaken, meaning that interviews should not be overheard by others and, where possible, undertaken in a private space where the individual and interviewer are not visible to others (with chaperones of the same gender). This will, in part, depend on the preference of the individual as some participants may prefer to stay in a public space.
4. Ensuring confidentiality of all data obtained from participants so that details about mental health conditions do not become known to people outside the research team.
5. Working with the Community Advisory Board to address stigma as part of the HOPE activities.

8 SOPs FOR SAFEGUARDING CONCERNS IDENTIFIED BY HOPE PROJECT

8.1 DEFINITIONS OF POTENTIAL HARMS

We have identified the following potential harms that could be experienced by people who are homeless, including those with severe MHCs, and require a safeguarding approach from the HOPE project. See Table 1 for the potential safeguarding concerns and their definitions.

Table 1: Definition of potential harms requiring a safeguarding response

	Potential harm	Definition
1	Chaining, restraint or seclusion	When a person is deprived of their liberty (freedom) in some way; for example, by being chained or tied up or locked in a room or in some other way restrained.
2	Suicidal behaviour or self-harm	An attempt by an individual to end their life (attempts to die by suicide) or harm themselves non-accidentally even if there is no intent to die.
3	Sexual exploitation, abuse or harassment	<u>Sexual abuse</u> is an actual or threatened physical intrusion of a sexual nature, whether by force or under unequal or coercive conditions. Sexual abuse includes sexually touching a person without their consent (e.g. groping or forced kissing). Rape is a form of sexual assault involving penetration of the vagina, anus or mouth.<br><br><u>Sexual exploitation</u> is any actual or attempted abuse of a position of vulnerability (e.g. mental illness), differential power or trust for sexual purposes. Sexual exploitation includes child sexual exploitation, exploitation of person with psychosis to be involved in sexual activities, project workers exchanging sex for money or goods or services.<br><br><u>Sexual harassment</u> includes sexually offensive comments, unwanted sexual advances, requesting sexual favours, gesturing or making sexual remarks about someone's body, clothing or appearance.
4	Physical abuse	Slapping, smacking, hitting, punching, kicking or spitting at someone or beating or harming the person with an implement (e.g. stick, knife).
5	Severe physical neglect or ill-health	Due to lack of basic needs and access to care, the person's health and/or nutritional status are at severe risk without intervention.
6	Neglect of children who live on the streets	Failure or absence of a caregiver to provide needed food, shelter, clothing, medical care, or supervision to the degree that a child's health (including nutritional status), safety, and well-being are at risk.
7	Violence or aggressive behaviour towards others	In the context of inadequate treatment and support, a minority of persons with severe MHCs may become violent or aggressive to others.
8	Coercion to take treatments or engage with services	Person is forcibly given treatment against their will. This could be medication or religious or traditional treatments or forcible removal to a service.
9	People trafficking	The children of people who are homeless, including those with severe mental health conditions, could be vulnerable to being trafficked by criminals.
10	Drug dealing or criminal activities	The person may be involved in criminal activities or dealing drugs.
11	Labour exploitation	The person may be exploited for their labour without remuneration or safe working conditions.

8.2 IDENTIFICATION OF HARMS

Harms against people who are homeless, with or without MHCs, may be identified through direct observation or through disclosures from the person themselves or reports from others. The most relevant means of identification of these harms in the context of HOPE are as follows:

Type of harm	Identification
Restraint, chaining or seclusion	Observed in a community setting, religious or traditional healer setting, social care facility or in health facilities.
Suicidal behaviour or self-harm	Reported by the person who is homeless (whether or not they have a diagnosis of a severe MHC) in the context of research activities, or during community case detection or during help-seeking at healing sites, social care facilities or in health facilities.
Sexual abuse, harassment or exploitation; Physical abuse; Severe physical health or neglect; Labour exploitation	Directly observed or reported by the person or local community members in the context of research activities, or during community case detection or during help-seeking at healing sites, social care facilities or in health facilities.
Violence or aggression towards others; involvement in drug dealing or criminal activities	Directly observed or reported by local community members in the context of research activities, or during community case detection or during help-seeking at healing sites or in health facilities.
Neglect of children who live on the streets; People Trafficking	Directly observed or reported by the person or local community members in the context of research activities, or during community case detection.
Coercion to take treatment or engage with services	Directly observed or reported by the person with severe MHC in the context of research activities or during help-seeking at healing sites or in health facilities.

8.3 ACTIONS TO BE TAKEN UPON IDENTIFICATION OF SAFEGUARDING CONCERNS

Flowcharts summarising the required response are presented in the Appendix.

The researchers will provide all participants with contact details for support and advocacy services as part of the recruitment process.

8.3.1 Chaining, restraint or seclusion in public settings

See flowchart 1. Note, for restraint or seclusion taking place on the premises of an organisation, refer to flowchart 6.

- Chaining, restraint or seclusion should be considered an emergency requiring action on the same day.
- The project-linked person will seek to understand why the person has been chained, restrained or secluded, and by whom, and explain the need for mental health care.
- The project-linked person will communicate with the project mental health focal person to plan how and where mental health care can be accessed.

- If the person who is chained, restrained or secluded is willing to attend for mental health care, the project-linked person will work with the health extension worker and/or community police to convey the person to that facility.
- If the person who is chained, restrained or secluded refuses to attend for mental health care, the project-linked person will work with the community police to convey the individual to mental health care.
- If a hospital admission is needed, the project mental health focal person will report this as a Serious Adverse Event.
- The project mental health focal person will co-ordinate with HOPE team and seek to ensure that the person has received care, is no longer restrained in the community and has ongoing mental health support.

8.3.2 Sexual abuse, exploitation or harassment

See flowchart 2.

- The project-linked person will first and foremost be supportive, take the allegations seriously and ensure privacy.
- If there is any concern that the situation is not safe, e.g. because a perpetrator of sexual assault is still in the vicinity, the project-linked person will re-locate to a safe place, with the affected individual if possible. A safe place means anywhere out of the vicinity or could be a government health facility. If that is not possible, the interview will stop and the project-linked person will inform the HOPE project safeguarding focal person immediately.
- The project-linked person will not force the person to speak about their experiences. They will be trained in principles of Psychological First Aid and will use these skills if the person is distressed. The project-linked person will ask about potential supports for the person and seek to mobilise support, with the person's permission.
- If the person reports sexual abuse, the project-linked person will encourage and support attendance at Ghandi hospital one-stop service for further assessment on the same day.
- The project-linked person will explain that they will need to speak to their seniors but that information will be kept confidential.
- Information about potential supports and places to go for help will be given verbally. If willing, the person will be supported to access these supports e.g. with provision of transport and being accompanied by a project worker.
- The HOPE safeguarding focal person should be informed on the same day and will communicate with the PIs to determine whether a sexual assault has taken place and whether a **Serious Adverse Event (SAE)** should be reported.
- The project mental health focal person should also be informed so that any necessary mental health support can be arranged, regardless of whether or not the person has a severe MHC.
- If a severe MHC is suspected, the project-linked person will liaise with the project mental health focal person so that an urgent assessment of decision-making capacity can be made.
- If the person wishes to report the incident to the appropriate authorities (e.g. Women and Social Affairs; police), the project will facilitate (e.g. with transport, accompanying the person), as needed.
- If the person has no MHC or if they have a severe MHC but are assessed to have decision-making capacity, If they wish not to report the incident, this will be respected.

If the person has a severe MHC and lacks decision-making capacity and does not wish to report the incident, the PIs will be informed. An urgent risk assessment will be conducted to determine whether to report the incident against the person's wishes. The decision will be recorded with justification.

8.3.3 Physical abuse or severe ill-health

Flowchart 3.

- If there is any concern that the situation is not safe, the project-linked person will re-locate to a safe place, with the affected individual if possible. A safe place means anywhere out of the vicinity or could be a government health facility. If that is not possible, the interview will stop and the project-linked person will inform the HOPE safeguarding focal person immediately.
- If someone is at immediate risk of serious harm of physical abuse, for example being threatened or attacked with a weapon, or if the project-linked person found that someone had been seriously injured due to physical abuse, or even killed, then the research team would inform the police in order to take urgent action and immediately report the incident to the HOPE safeguarding focal person and/or one of the HOPE PIs.
- If physical abuse is being perpetrated by the police, the project-linked worker will immediately contact the HOPE safeguarding focal person and/or one of the HOPE PIs. It is essential that the project-linked worker should prioritise their own safety. The incident will be followed up by the HOPE PIs and safeguarding officer, including a discussion about whether the police officer can or should be reported. In any case, HOPE will engage with the police via the community advisory board and directly to promote approaches that uphold the human rights of people who are homeless, including those who have a severe MHC.
- If there are serious concerns about the person's physical health e.g. due to physical abuse, accidental injury or because of medical illness or severe nutritional deficiency, the project-linked worker will urgently contact the family health team or health extension worker to arrange access to emergency health care.
- The HOPE safeguarding focal person should be informed on the same day and will communicate with the PIs to determine whether a **Serious Adverse Event (SAE)** should be reported.
- If a severe MHC is suspected, the project-linked person will liaise with the project mental health focal person so that an urgent assessment of decision-making capacity can be made.
- If the person wishes to report the incident to the appropriate authorities, the project will facilitate (e.g. with transport, accompanying the person), as needed.
- If the person has no MHC or if they have a severe MHC but are assessed to have decision-making capacity, if they refuse health care or decline to report the incident, this decision will be respected. In that case, the person will be provided with information about how and where they can seek help should they want it.
- If the person has a severe MHC and lacks decision-making capacity and does not wish to access healthcare or report the incident, the PIs will be informed. An urgent risk assessment will be conducted to determine whether to override person's wishes. The decision will be recorded with justification.

8.3.4 Suicidal behaviour or self-harm

Flowchart 4.

- The project-linked person will first and foremost be supportive and respectful towards the person.
- The project-linked person will stay with the person and remove potential means of suicide in the vicinity.
- They will contact the family health team or health extension worker and support urgent attendance of the person for a mental health assessment in the health centre or other health facility.
- The project-linked person will inform the HOPE safeguarding focal person who will complete reporting for an Serious Adverse Event (SAE) if relevant.
- The project-linked person will contact the HOPE mental health focal person to plan how and where mental health care can be accessed.
- If the person refuses mental health care, the HOPE mental health focal person will arrange for urgent assessment of decision-making capacity, suicide risk and mental health needs.
- If the person is assessed as having a high suicide risk and lacks decision-making capacity and refuses mental health care, project staff will co-ordinate with the community police to convey to mental health care.

8.3.5 Violent or aggressive behaviour

Flowchart 5.

- If there is any concern that the situation is not safe, the project-linked person will re-locate to a safe place, the interview will stop and the project-linked person will inform the HOPE safeguarding focal person immediately.
- The project-linked person should prioritise their own safety.
- If it is safe to do so, they should speak calmly and respectfully to the person throughout and encourage others to do the same.
- If a member of the public is injured, they should support them to access health care by arranging transport.
- If the person appears to have a mental health condition, work with community police to arrange urgent transfer to mental health services.
- If there is no apparent mental health condition, inform the community police so that they can take appropriate action.
- The project-linked person will then report to the HOPE safeguarding focal person who will determine whether a Serious Adverse Event has occurred i.e., if there is violent behaviour causing injury or arrest/imprisonment.

- If the person has a mental health condition, the HOPE mental health focal person will be informed. The HOPE mental health focal person will help to co-ordinate access to mental health services and will report a Serious Adverse Event if hospitalisation is required.

8.3.6 Coercive treatment, restraint or abuse on the premises of facilities or healing sites

Flowchart 6.

- Any of the above safeguarding concerns could take place on the premises of an organisation; for example, a health facility, social care facility, traditional or religious healing site, church, mosque or police station.
- In such situations, if there is any concern that the situation is not safe, the person working with the HOPE project should re-locate to a safe place and inform the HOPE safeguarding focal person immediately.
- If there is any immediate and serious threat to the person, inform the police immediately. However, if the police are the perpetrators, the project-linked person should first prioritise their own safety. They should then inform the HOPE safeguarding focal person and/or PIs immediately.
  - The incident will be followed up by the HOPE PIs and safeguarding focal person, including a discussion about whether the police officer can or should be reported.
  - Plans will be made to discuss with the multi-sectoral Community Advisory Board in each study area to plan the best way to tackle such practices and incidents on an ongoing basis. Usually this will be on a 6-monthly basis, but if needed the senior research team will convene a sub-committee to address a specific issue.
- If action is safe, the project-linked person will follow the relevant flow chart, depending on the nature of the safeguarding concern.
- The HOPE safeguarding focal person should be contacted on the same day and will determine whether an SAE needs to be reported.

8.3.7 People Trafficking or involvement in Drug Dealing or Criminal Activities

Any concerns about possible people trafficking activities, drug dealing or criminal activities that put others at immediate risk will be reported directly by project-linked person to the HOPE safeguarding focal person and from there to the PIs. This senior management team will then report any concerns to the appropriate authorities.

8.3.8 Neglect of children living on the street

Any concerns about serious neglect of children (under 15 years of age) living on the street will be reported to the Women and Social Affairs Office and local health team.

9 APPENDIX: FLOW CHARTS FOR RESPONSES TO SAFEGUARDING CONCERNS

1. Chaining, restraint or seclusion in the community

Identified by person linked to the HOPE project

↓

Seek to identify why the person is chained, restrained or secluded and explain the need for mental health care

↓

Communicate with the project mental health focal person to plan how and where mental health care can be accessed

As emergency

↓

If person is **willing to receive** mental health care, work with health extension worker or community police to support the person to access the facility

↓

If the person **refuses to receive** mental health care, involvement of community police is essential

↓

Project **focal mental health person** to complete SAE report if person is admitted to hospital

↓

<u>Follow-up:</u> Project focal mental health person to co-ordinate with HOPE team and seek to ensure that the person has received care, is no longer restrained in the community and has ongoing mental health support

2. Sexual abuse, exploitation or harassment

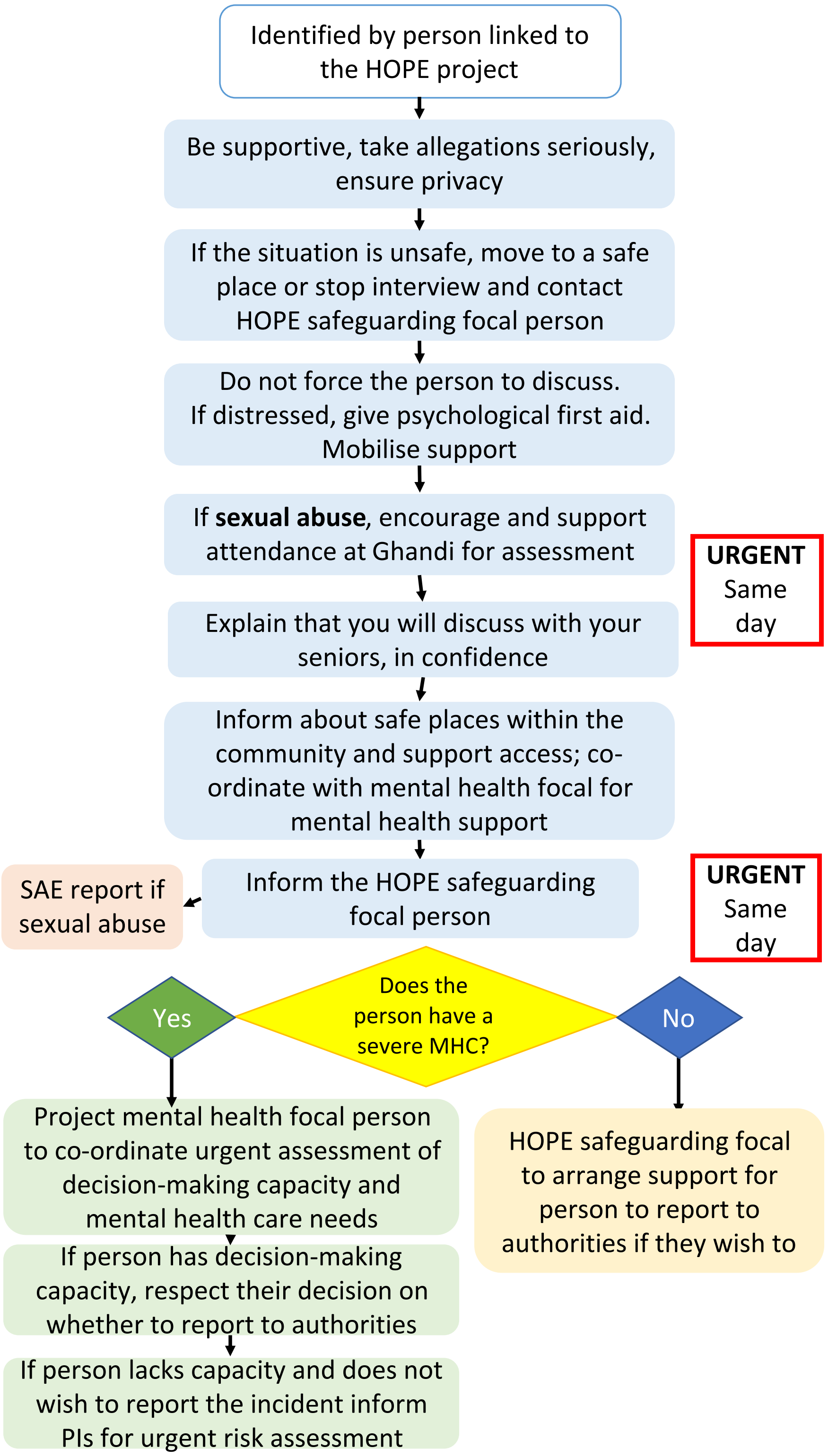

3. Physical abuse or serious physical health concerns

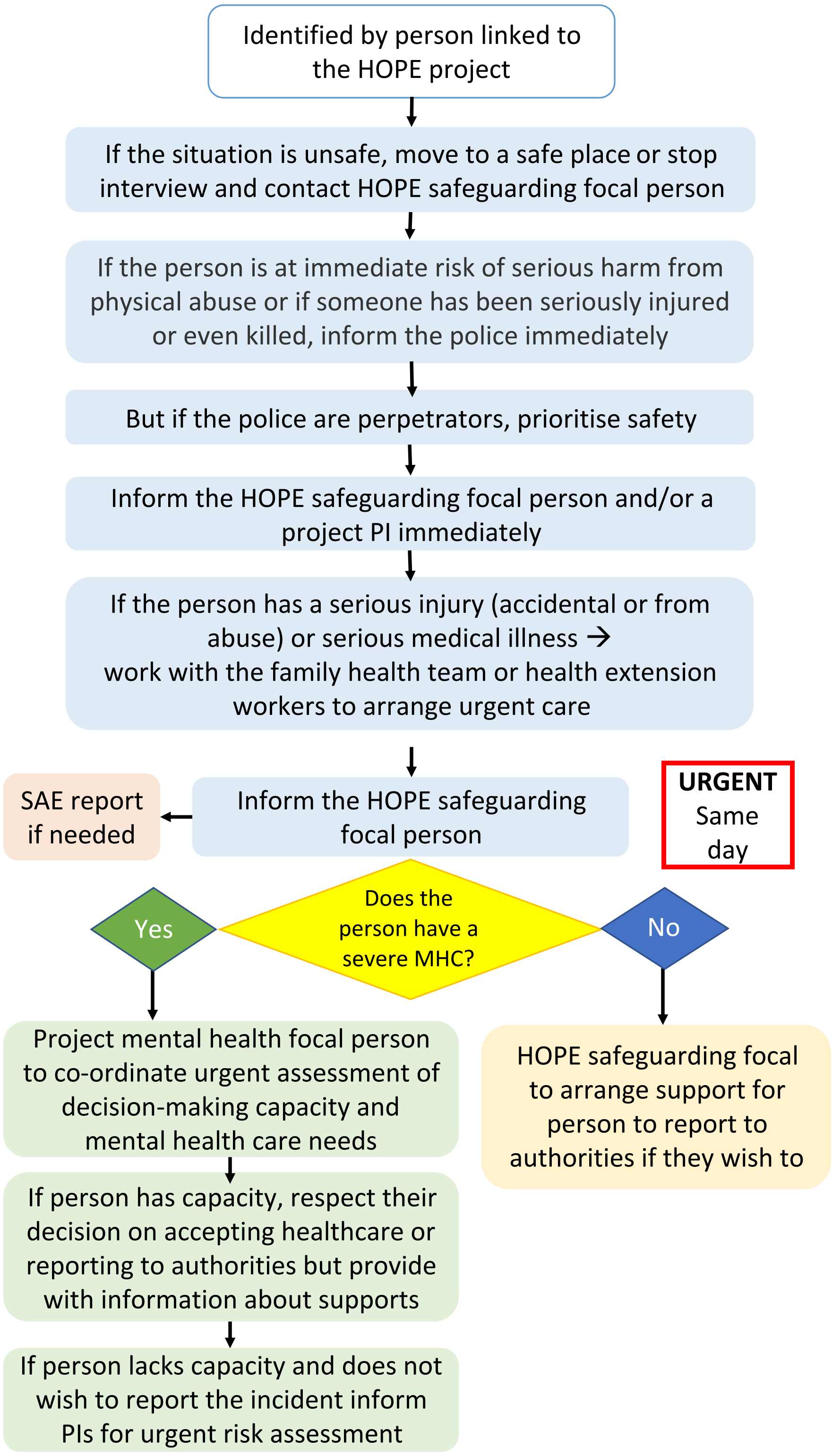

4. Suicidal behaviour or selfharm

Identified by person linked to the HOPE project

↓

Be supportive and respectful to the individual

↓

Stay with the person and remove potential means of harm

↓

Work with family health team or health extension worker to support attendance for an urgent assessment at the health centre

↓

Inform HOPE safeguarding focal person and HOPE mental health focal person → **URGENT** same day → SAE report for suicide attempt

↓

If the person **refuses** mental health care, HOPE mental health focal person to co-ordinate urgent assessment of decision-making capacity, suicide risk and mental health care needs

↓

If person has high suicide risk and lacks decision-making capacity and refuses mental health care, work with community police to convey to mental health care → Project **focal mental health person** to complete SAE report if person is admitted to hospital

5. Violent or aggressivebehaviour

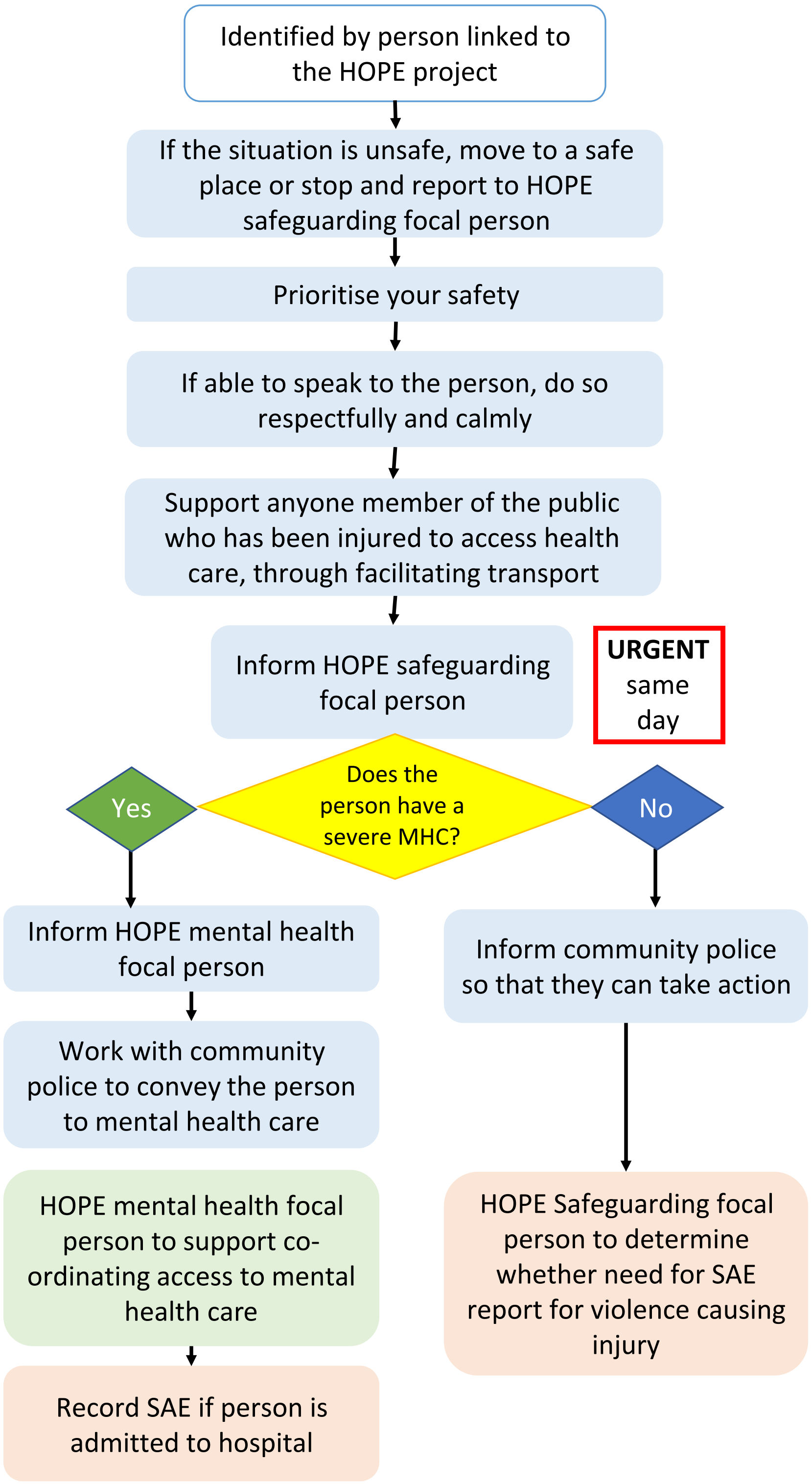

6. Coercive treatment, restraint or abuse on premises of health facilities, social care facilities, churches, healing sites or police stations

Identified by person linked to the HOPE project

↓

If the situation is unsafe, move to a safe place or stop and report to HOPE safeguarding focal person

↓

If there is any immediate and serious threat to the person, inform the police immediately

↓

If the police are the perpetrators, do not try to intervene

↓

Inform HOPE safeguarding focal person and/or a project PI immediately → **URGENT** On the same day → SAE report if relevant

↓

If safe, follow the appropriate flow chart depending on the nature of the safeguarding concern
